# Supplementary material for: Continuously processing waste lignin into high-value carbon nanotube fibers
Source: Nat Commun. 2022 Sep 30;13:5755. doi: 10.1038/s41467-022-33496-2 (PMC9525656; doi:10.1038/s41467-022-33496-2)
Supplement: Supplementary file 1 — Supplementary Information [file 41467_2022_33496_MOESM1_ESM.pdf]

Supplementary Information

Continuously Processing Waste Lignin into High-value Carbon Nanotube Fibers

Fuyao Liu, Qianqian Wang, Gongxun Zhai, Hengxue Xiang*, Jialiang Zhou, Chao Jia*, Liping Zhu, Qilin Wu, Meifang Zhu*

State Key Laboratory for Modification of Chemical Fibers and Polymer Materials, College of Materials Science and Engineering, Donghua University, Shanghai 201620, China

***Email:** hengxuexiang@dhu.edu.cn; jiachao0806@dhu.edu.cn; zmf@dhu.edu.cn

This file includes:

Supplementary Table 1 to 10

Supplementary Figure 1 to 28

Supplementary Reference 1 to 64

Supplementary Tables

Supplementary Table 1. Comparison of the structures and morphologies of our prepared CNTs with those of other reported CNTs.

Biomass	Carbon source type	Continuous preparation	Synthesis temperature (°C)	I_G/I_D	CNT type	Micro morphology	Reference
Lignin	Biomass	Yes (120 m h^{-1})	1400	3.84	MWNTs	CNT aerogels	Our work
Eucalyptus oil	Biomass	No	850	3.3	SWNTs/ MWNTs	CNT powders	1
Turpentine oil	Biomass	No	700	0.93	MWNTs	CNT arrays	2
Sesame oil	Biomass	No	900	0.98	MWNTs	CNT arrays	3
Grass	Biomass	No	600	2.0	MWNTs	CNT arrays	4
Plant	Biomass	No	> 600	-	MWNTs	CNT powders	5
Poplar leaves	Biomass	No	450	0.79	MWNTs	CNT powders	6
Methane	Chemicals	Yes	1200	1.2	MWNTs	CNT aerogels	7
Hexane	Chemicals	Yes	1150-1500	3.36-10.43	MWNTs	CNT aerogels	8
Ethanol	Chemicals	Yes	1150–1300	3.74	MWNTs	CNT aerogels	9
Toluene	Chemicals	Yes	1200	1.78-14.28	MWNTs	CNT aerogels	10
Acetylene	Chemicals	Yes	-	1.22	MWNTs	CNT arrays	11
Toluene	Chemicals	Yes	1150	0.94	MWNTs	CNT aerogels	12
Xylene/dichlorobenzene	Chemicals	No	800	1.11	MWNTs	CNT arrays	13
Methane	Chemicals	Yes	1175	3.61	MWNTs	CNT aerogels	14
Methane	Chemicals	Yes	1200	7.7-14.3	SWNTs/ DWNTs	CNT aerogels	7
Toluene	Chemicals	Yes	1200	1.92-33.33	SWNTs	CNT aerogels	10
Acetylene/ Ethylene	Chemicals	Yes	1175	18.52-69.87	SWNTs/ DWNTs	CNT aerogels	14
Ethanol	Chemicals	Yes	1000	> 30	SWNTs	CNT aerogels	15
Carbon monoxide	Chemicals	Yes	800-1050	10-440	SWNTs	CNT films	16
Acetone	Chemicals	Yes	1200	10-	SWNTs	CNT aerogels	17

				78.69			
Methane	Chemicals	Yes	1200	> 60	SWNTs/ DWNTs	CNT aerogels	18
Carbon monoxide	Chemicals	Yes	880	224	SWNTs	CNT films	19

Note: SWNTs: single-walled carbon nanotubes; DWNTs: double-walled carbon nanotubes; MWNTs: multi-walled carbon nanotubes.

Supplementary Table 2. Assignments of ^{13}C-^{1}H cross signals in the HSQC spectra of lignin.

**Label**	***δ*C/*δ*H (ppm)**	**Assignments**
B_{β}	53.5/3.07	Cβ-Hβ in β-β resinol (B)
OCH_3	56.4/3.70	C-H in methoxyls
Aγ	59.9/3.35-3.80	Cγ-Hγ in β-O-4 substructures (A)
Bγ	71.2/3.82-4.18	Cγ-Hγ in β-β resinol (B)
Aα	71.8/4.86	Cα-Hα in β-O-4 unit (A)
Aβ(G)	83.4/4.38	Cβ-Hβ in β-O-4 linked to G (A)
Bα	84.8/4.66	Cα-Hα in β-β resinol (B)
S2,6	103.9/6.70	C2,6-H2,6 in syringyl units (S)
S'2,6	106.3/7.32	C2,6-H2,6 in oxidized S units (S')
G2	110.8/6.97	C2-H5 in guaiacyl units (G)
G5	114.5/6.70	C5-H5 in guaiacyl units (G)
G6	119.0/6.78	C6-H6 in guaiacyl units (G)

Supplementary Table 3. Simulated analysis result of lignin molecule from ChemDraw.

**Item**	**Result**
Chemical formula	$C_{205}H_{234}O_{81}$
Exact mass	3991.4
Molecular weight	3994.0
Elemental analysis	C: 61.65, H: 5.91, O: 32.45

Supplementary Table 4. Main pyrolysis products of lignin at 800°C from PY-GCMS.

Number	Time (s)	Area (%)	Formula	CAS Number	Remark
1	1.70	23.90			Multi-component peak
2	1.78	2.49	C_5H_8O	10229-10-4	
3	1.92	31.92			Multi-component peak
4	1.96	0.17	C_4H_6	590-19-2	Other
5	2.00	0.55	C_5H_8	78-79-5	Other
6	2.13	1.93	C_5H_6	542-92-7	
7	2.36	0.20	C_5H_6	542-92-7	Other
8	2.63	0.07	C_6H_{10}	513-81-5	Other
9	2.85	0.84	C_6H_8	592-57-4	Other
10	2.89	0.65	C_6H_8	96-39-9	Other
11	3.12	6.05	C_6H_6	71-43-2	
12	3.21	1.03	C_4H_4S	110-02-1	
13	4.76	5.74	C_7H_8	108-88-3	
14	4.84	0.56	C_5H_6S	616-44-4	Other
15	4.98	0.35	C_5H_6S	554-14-3	Other
16	6.40	0.50	C_8H_{10}	100-41-4	Other
17	6.54	1.53	C_8H_{10}	108-38-3	
18	6.70	0.16	C_6H_8S	632-15-5	Other
19	6.92	2.07	C_8H_8	100-42-5	
20	8.01	0.25	C_9H_{12}	620-14-4	Other
21	8.31	0.28	C_9H_{10}	98-83-9	Other
22	8.50	0.91	C_9H_{10}	611-15-4	Other
23	8.56	0.36	C_8H_6O	271-89-6	Other
24	8.66	0.81	C_6H_6O	108-95-2	Other
25	9.22	1.84	C_9H_8	95-13-6	
26	9.48	2.27	C_7H_8O	95-48-7	
27	9.84	0.88	C_7H_8O	108-39-4	Other
28	9.91	0.96	C_9H_8O	17059-52-8	Other
29	9.99	0.66	$C_8H_8N_2$	614-97-1	Other
30	10.07	0.89	$C_8H_{10}O$	576-26-1	Other
31	10.54	0.89	$C_{10}H_{10}$	2177-47-1	Other
32	10.63	2.22	$C_{10}H_{10}$	767-59-9	
33	11.01	1.84	$C_{10}H_8$	91-20-3	
34	11.13	0.15	C_8H_6S	270-82-6	Other
35	11.14	0.18	$C_9H_{10}N_2$	582-60-5	Other
36	11.21	0.31	$C_9H_{12}O$	2416-94-6	Other
37	12.25	1.09	$C_{11}H_{10}$	91-57-6	
38	12.43	0.70	$C_{11}H_{10}$	90-12-0	Other
39	13.09	0.16	$C_{12}H_{10}$	92-52-4	Other
40	13.37	0.18	$C_{12}H_{12}$	575-37-1	Other
41	13.51	0.18	$C_{12}H_{12}$	571-58-4	Other
42	13.62	0.18	$C_{12}H_{10}$	827-54-3	Other
43	13.86	0.44	$C_{12}H_8$	259-79-0	Other
44	14.09	0.13	$C_{13}H_{12}$	644-08-6	Other
45	15.50	0.31	$C_{11}H_{10}O$	7469-77-4	Other

Supplementary Table 5. Composition and proportion of small molecule products from lignin pyrolysis at 800°C (Split the peaks at retention times of 1.70 and 1.92 in Supplementary Table 4 and Supplementary Fig. 8).

Number	Time (s)	Area (%)	Formula	CAS Number	Remark
1	1.70	23.90	/	/	Multi-component peak
3	1.92	31.92	/	/	Multi-component peak
Total		55.82			
**M/Z**	**Area (%)**	**Area (%)**	**Formula**	**CAS Number**	**Remark**
28	31.26	17.45	CO	630-08-0	
44	13.00	7.26	CO_2	124-38-9	
18	48.54	27.09	H_2O	7732-18-5	
16	7.19	4.02	CH_4	74-82-8	
Total	100	55.82			

Supplementary Table 6. Comparison of thermal conductivity of our CNT films and CNT films with similar characteristics prepared by the similar method, other biomass-derived carbon materials, as well as common metals.

**Materials**	**Thermal conductivity ($W\ m^{-1}\ K^{-1}$)**	**Density ($g\ cm^{-3}$)**	**Reference**
Lignin-CNT films	33.21	0.82	Our work
PMMA/CNT	3.44	1.18	20
Polycarbonate/CNT	1.27	1.2	21
Lignin-based carbon foams	0.75	0.68	22
Lignin-based carbon fibers	24	2.189	23
Lignin-based carbon fibers	1.8	2	24
Lignin wood	0.23	1.2	25
Lignin aerogels	0.06	2.5	26
Ethanol-CNT films	20.91-458.58	0.37-1.59	27
Silver (Ag)	419	10.49	28
Copper (Cu)	385	7.76	28
Aluminum (Al)	210	2.7	28
Iron (Fe)	76.2	7.87	28

Supplementary Table 7. Comparison of tensile strength and electrical conductivity of lignin-based CNT fibers with other carbon-based fibers and metal materials.

**Materials**	**Tensile strength (GPa)**	**Electrical conductivity ($S\ m^{-1}$)**	**Reference**
Lignin-CNT fibers	1.33	1.19×10^{5}	Our work
Biomass-derived carbon fibers	0.088	1.03×10^{4}	29
Biomass-derived carbon fibers	0.369	1.91×10^{4}	30
Biomass-derived carbon fibers	0.351	1.41×10^{4}	31
Biomass-derived carbon fibers	0.129	5×10^{3}	32
Biomass-derived carbon fibers	1.648	1.85×10^{4}	33
Biomass-derived carbon fibers	0.763	2×10^{3}	34
Biomass-derived carbon fibers	0.59	1×10^{3}	35
Biomass-derived carbon fibers	0.57	3×10^{3}	35
Array CNT fibers	0.656	4.08×10^{4}	36
Array CNT fibers	1.408	5.84×10^{4}	37
Array CNT fibers	1.408	2.39×10^{5}	37
Array CNT fibers	1.90	6×10^{4}	38
Array CNT fibers	1.91	4.1×10^{4}	39
Array CNT fibers	0.389	1.05×10^{5}	11
FCCVD CNT fibers	1.0	1.43×10^{5}	40
FCCVD CNT fibers	0.38	4.6×10^{5}	41
FCCVD CNT fibers	0.32	2.0×10^{6}	42
FCCVD CNT fibers	0.36	1.27×10^{5}	9

FCCVD CNT fibers	4.34	2.05×10^{6}	9
FCCVD CNT fibers	0.27	1.657×10^{5}	43
FCCVD CNT fibers	2.81	1.2×10^{6}	43
Wet-spun CNT fibers	0.116	5×10^{5}	44
Wet-spun CNT fibers	1.0	2.9×10^{6}	45
Wet-spun CNT fibers	1.0	5.0×10^{6}	45
Wet-spun CNT fibers	2.4	8.5×10^{6}	46
Wet-spun CNT fibers	4.2	1.09×10^{7}	47
Hexcel (AS4)	4.27	6.5×10^{4}	48
Cytec (T300)	3.75	5.56×10^{4}	48
Toray (T300)	3.53	5.9×10^{4}	48
Toray (T1000G)	6.37	7.14×10^{4}	48
Toray (M55J)	4.02	1.25×10^{5}	48
Cytec (K-800X)	2.34	8.83×10^{5}	48
Cytec (K-1100)	3.10	9.09×10^{5}	48
Silver (Ag)	0.14	6.3×10^{7}	28
Copper (Cu)	0.21	5.8×10^{7}	28
Aluminum (Al)	0.1	3.5×10^{7}	28
Iron (Fe)	0.54	1.0×10^{7}	28

Supplementary Table 8. Comparison of production rate of CNT fibers from lignin and fine chemicals.

Methods	Carbon source		Production rate (m h^{-1})	Reference
FCCVD	Biomass	Lignin	120	Our work
Array	Fine chemicals	Acetylene	60-600	49
Wet-spinning		Methane	300-540	7
FCCVD		Ethanol	120-1200	9
FCCVD		Methane	300	50
FCCVD		Butanol	300-480	51
FCCVD		Acetone	300-1800	52
FCCVD		Methane	330	53
FCCVD		Butanol	420-540	54
FCCVD		Acetone	450-540	55
FCCVD		Ethanol	600	56
FCCVD		Butanol	600	57
FCCVD		Toluene	900	41

Supplementary Table 9. Comparison of the amount of ferrocene we used for preparing lignin-based CNT fibers with other literatures.

**Carbon source (Carrier gas)**	**Ferrocene concentration in solution (g mL^{-1})**	**Mass fraction of ferrocene in solution (%)**	**Reference**
Lignin (Ar)	0.005	0.62	Our work
N-hexane (H_2+Ar)	0.007-0.013	-	58
Xylene+ dichlorobenzene (H_2)	0.1	-	13
Xylene (H_2+Ar)	0.05	-	59
Cyclohexane (H_2)	0.02	-	60
Toluene (H_2+Ar)	0.03	-	12
Ethanol (H_2+Ar)	-	0.1-3	61
Ethanol (H_2+N_2)	-	0.25-0.4	15
Toluene (H_2)	-	1-9.6	62
Methane (H_2)	-	0.31-0.89	7

Supplementary Table 10. Composition analysis of lignin.

**Analysis method**	**Component**	**Content (%)**
Proximate analysis	Moisture	2.3
	Ash	1.1
	Volatiles	59
	Fixed carbon	37.7
Ultimate analysis	Carbon	61.92
	Hydrogen	5.68
	Oxygen	31.92
	Nitrogen	≤0.05
	Sodium	≤0.005

Supplementary Figures

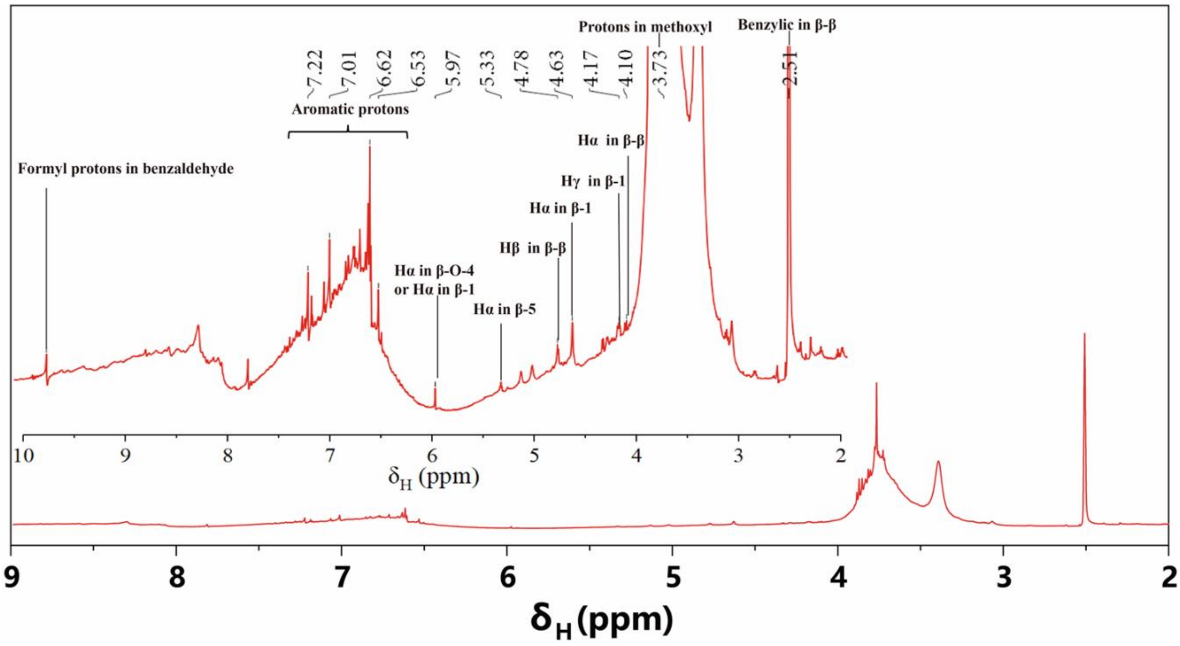

Supplementary Figure 1. Quantitative ^1H NMR spectrum of lignin.

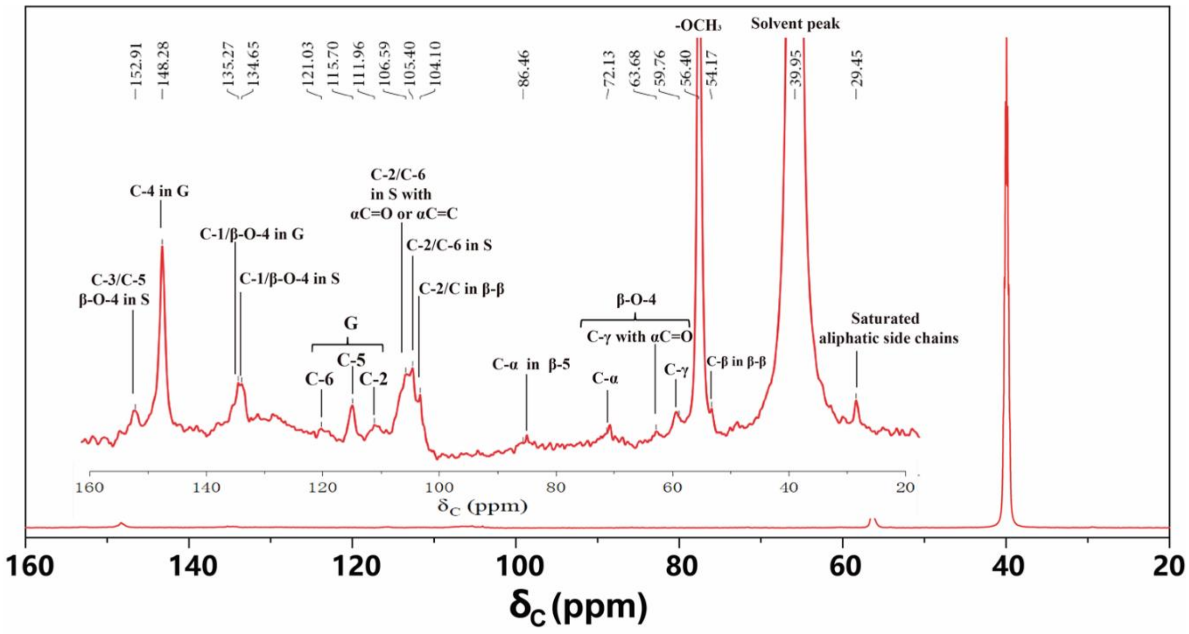

Supplementary Figure 2. Quantitative ^{13}C NMR spectrum of lignin.

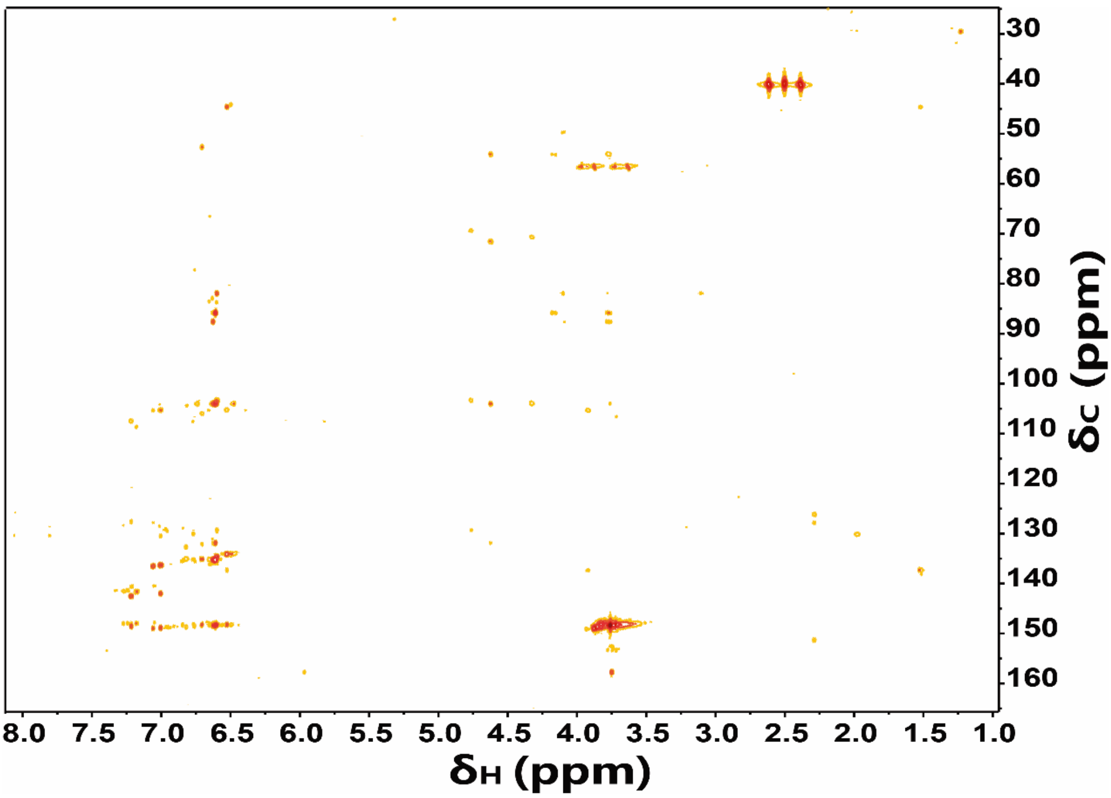

Supplementary Figure 3. HMBC spectra (600 MHz with a cryogenic probe, DMSO-d6) of lignin.

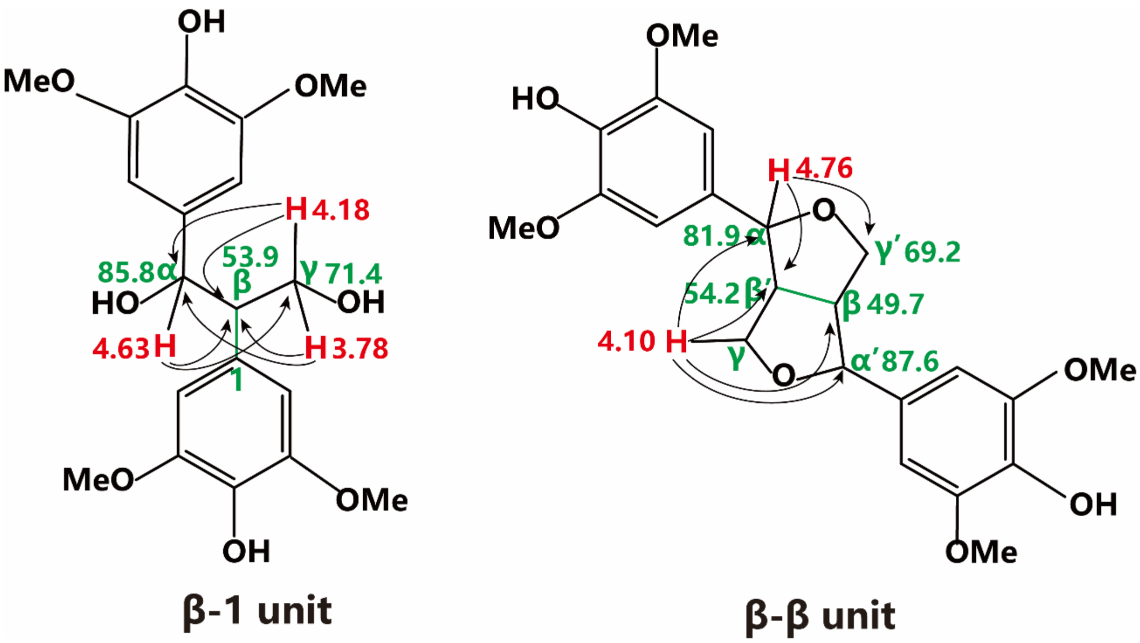

Supplementary Figure 4. Main linkages of lignin obtained from HMBC spectra.

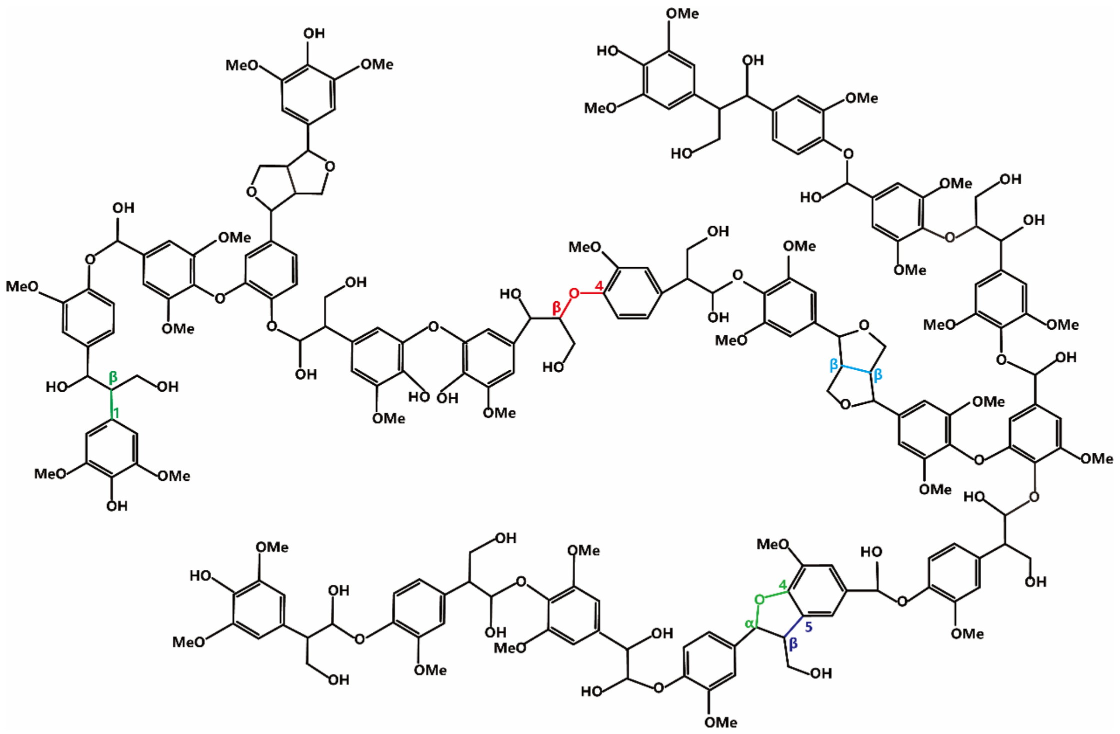

Supplementary Figure 5. Molecular structure of lignin deduced from the results of NMR.

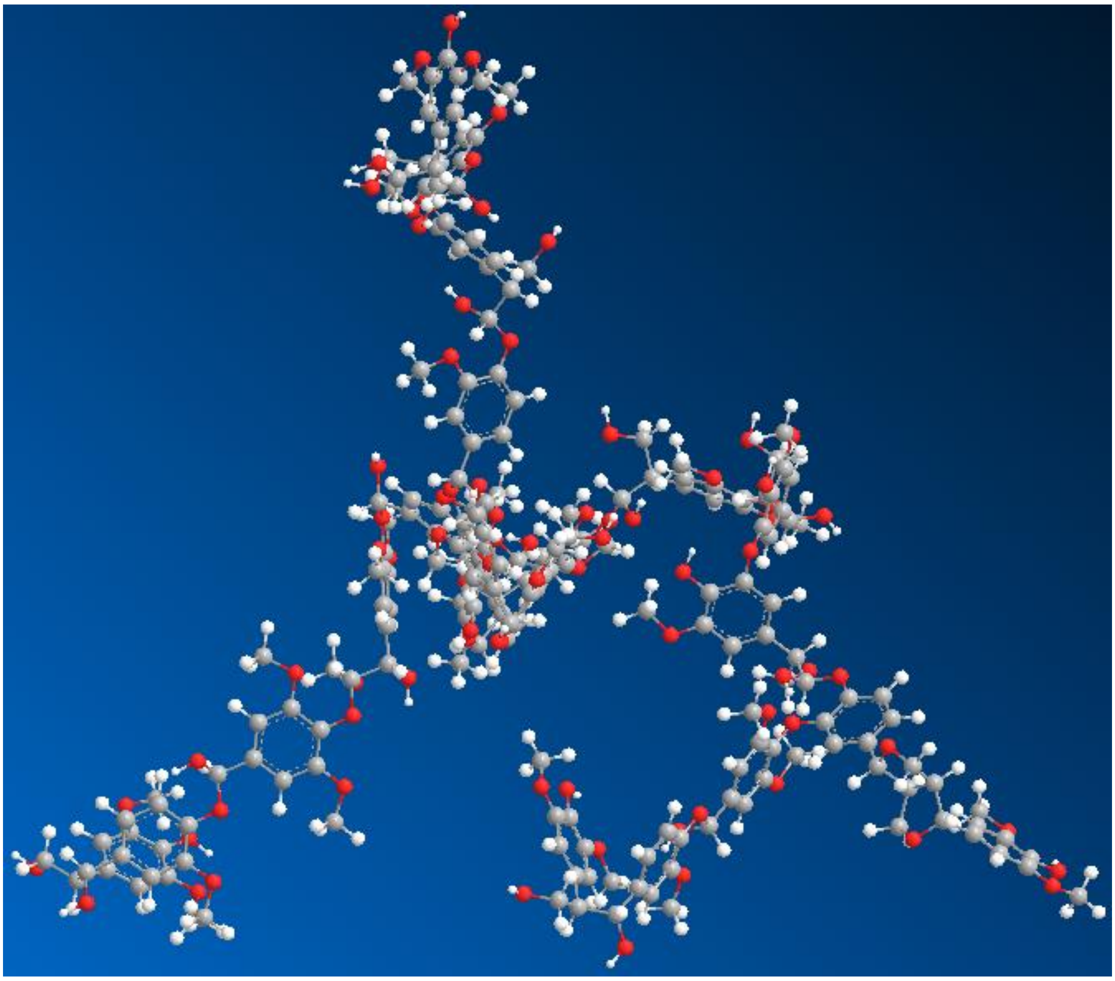

Supplementary Figure 6. Three dimensional structure of lignin molecule simulated by ChemDraw software.

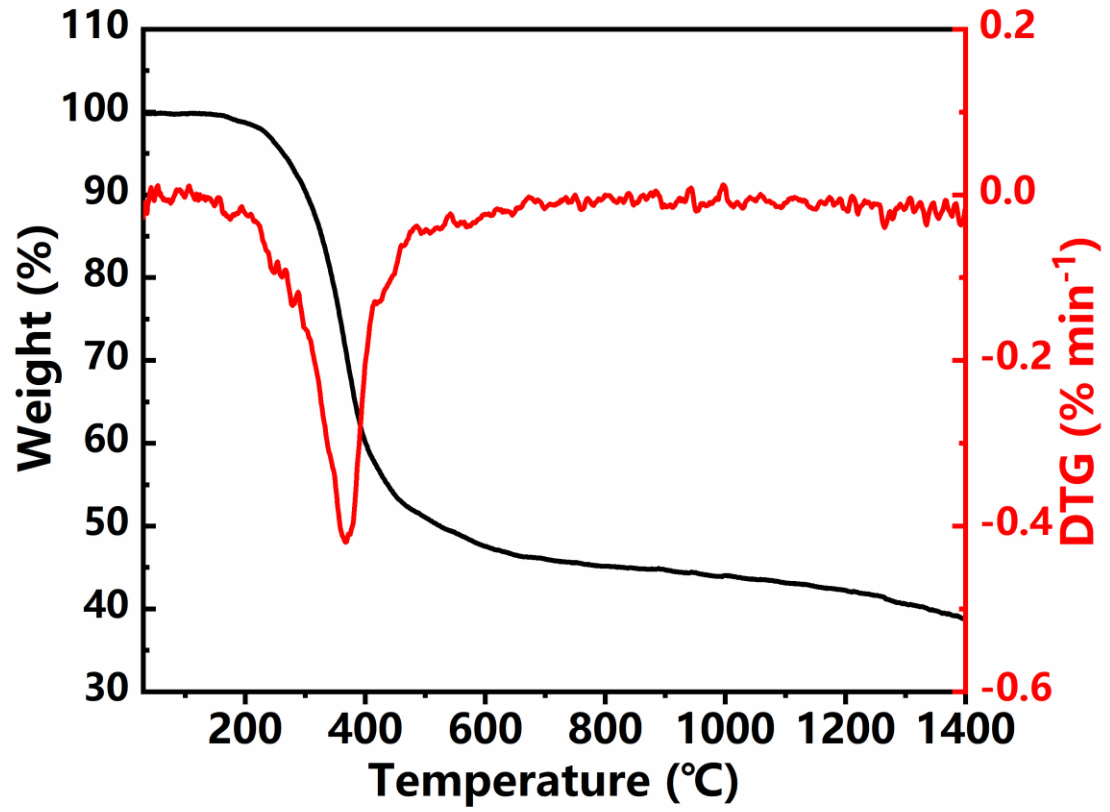

Supplementary Figure 7. Thermogravimetric (TG) analysis of lignin in a nitrogen atmosphere.

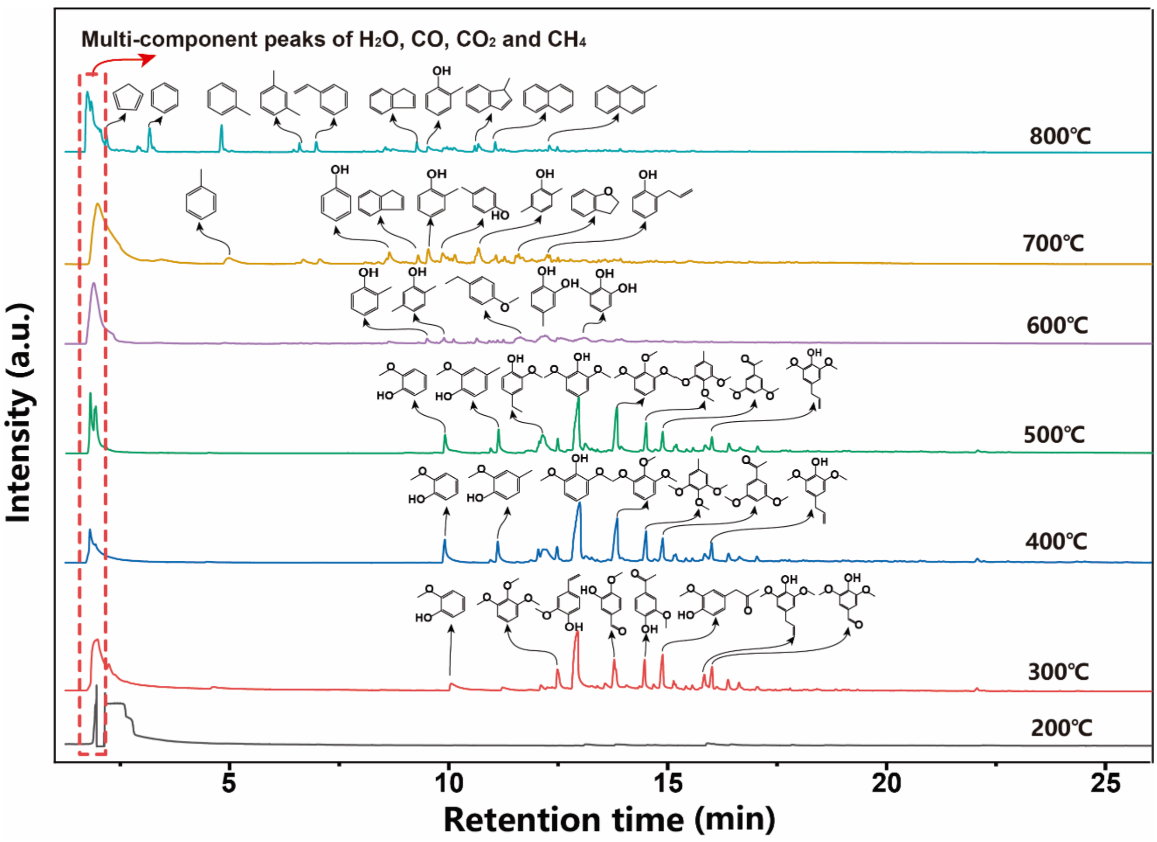

Supplementary Figure 8. Pyrolysis products of lignin at different temperatures obtained by pyrolysis gas chromatography mass spectrometry (PY-GCMS).

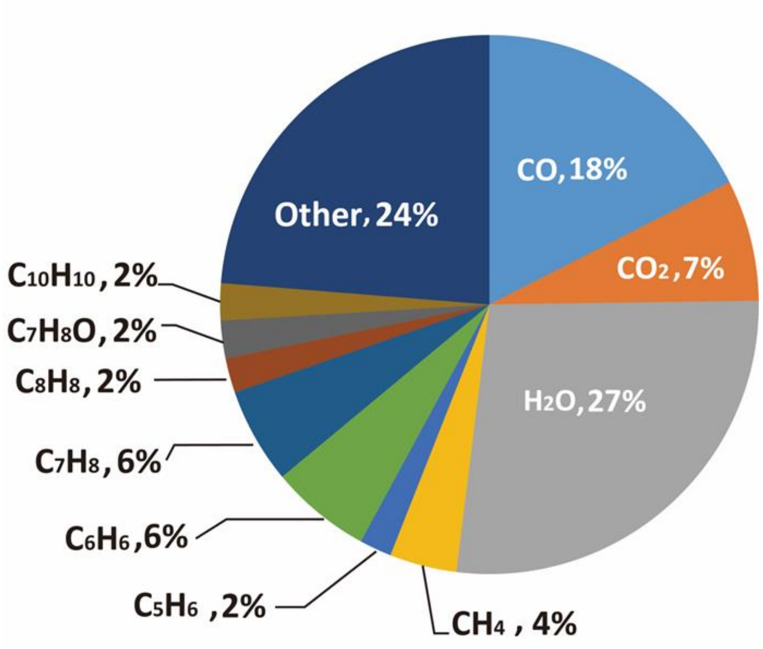

Supplementary Figure 9. Main pyrolysis products of lignin at 800°C from PY-GCMS.

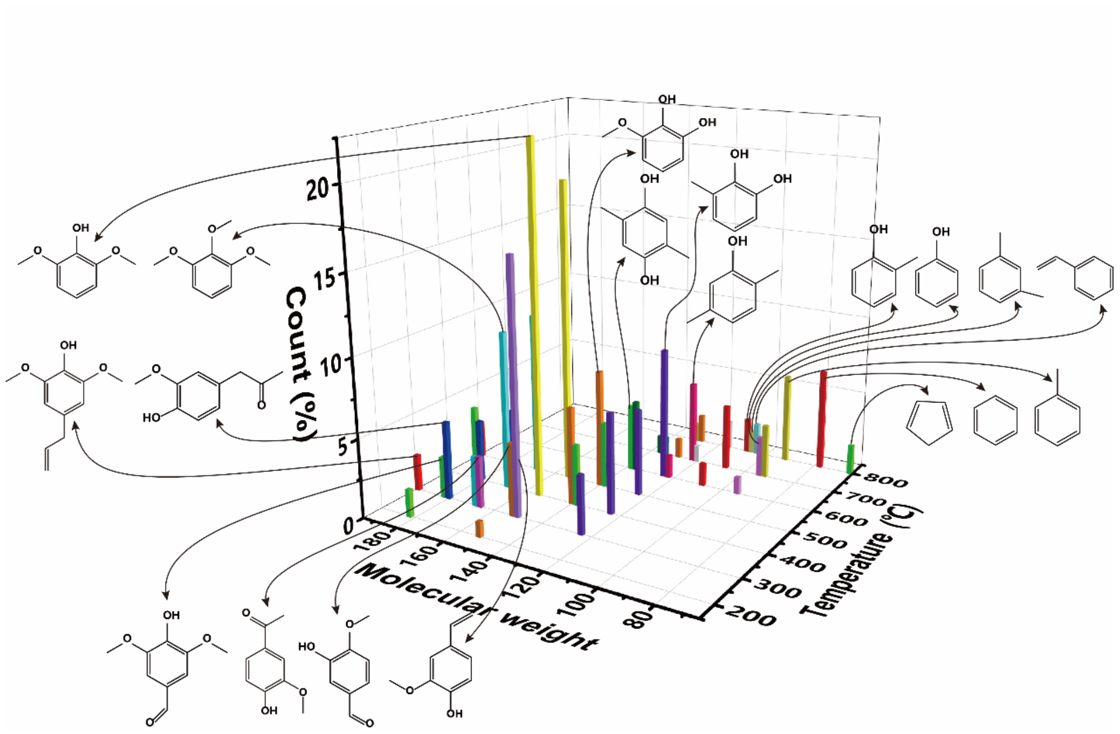

Supplementary Figure 10. Main pyrolysis products (aromatic hydrocarbon) of lignin in the temperature range of 200-800°C from PY-GCMS analysis.

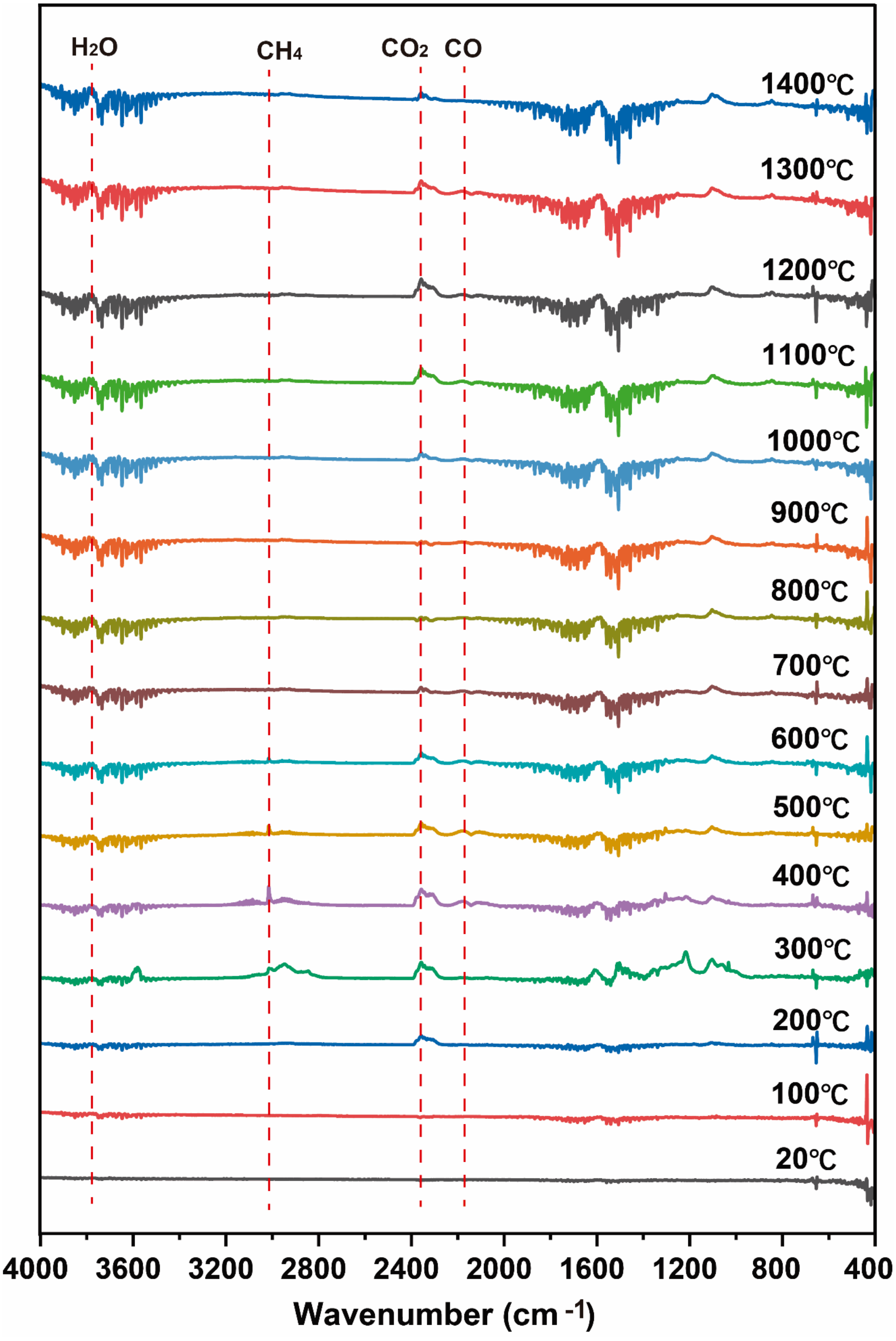

Supplementary Figure 11. FTIR spectra of pyrolysis products of lignin at 20-1400°C.

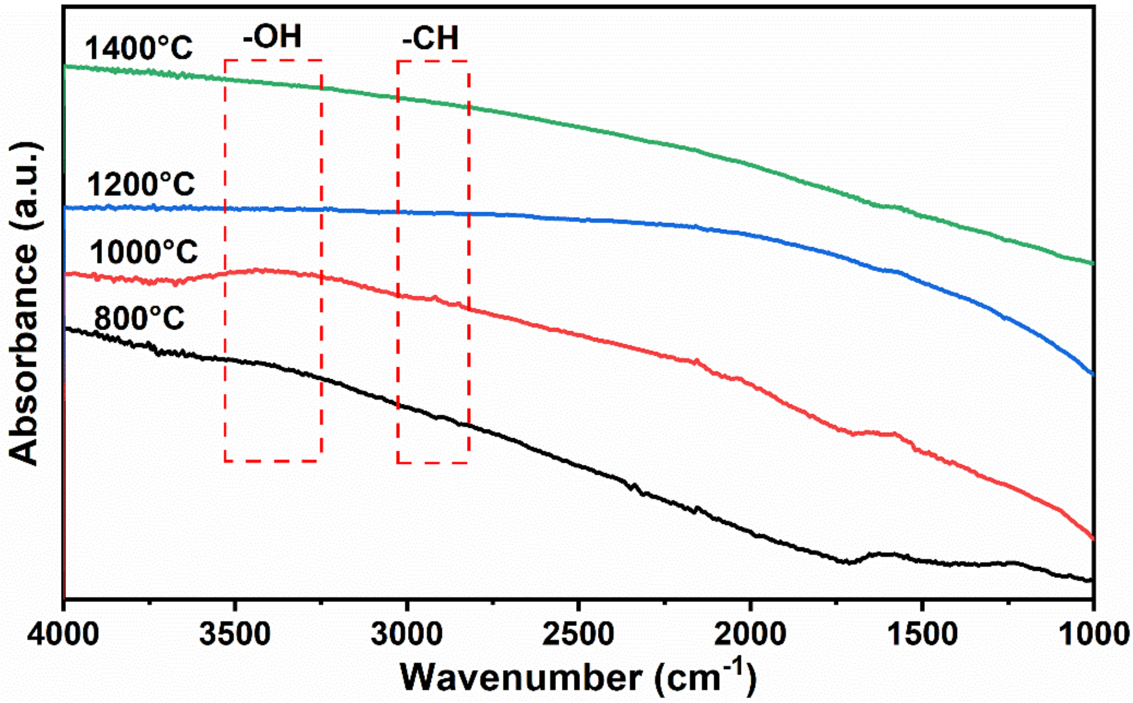

Supplementary Figure 12. FTIR spectra of lignin charcoal at 800-1400°C.

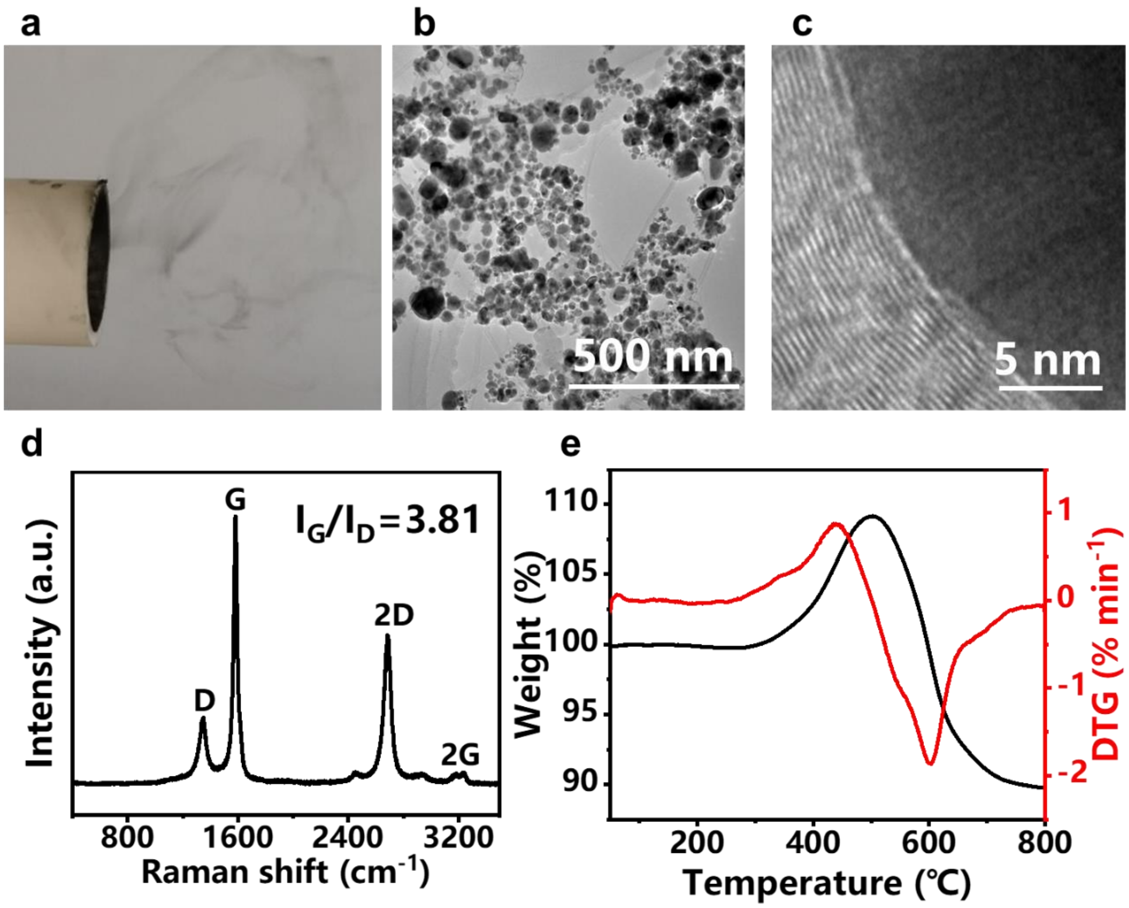

Supplementary Figure 13. Carbon nanospheres synthesis without sulfur. a Digital image and **b, c** TEM images of the carbon nanospheres. **d** Raman spectrum and **e** TGA of the carbon nanospheres prepared without thiophene addition.

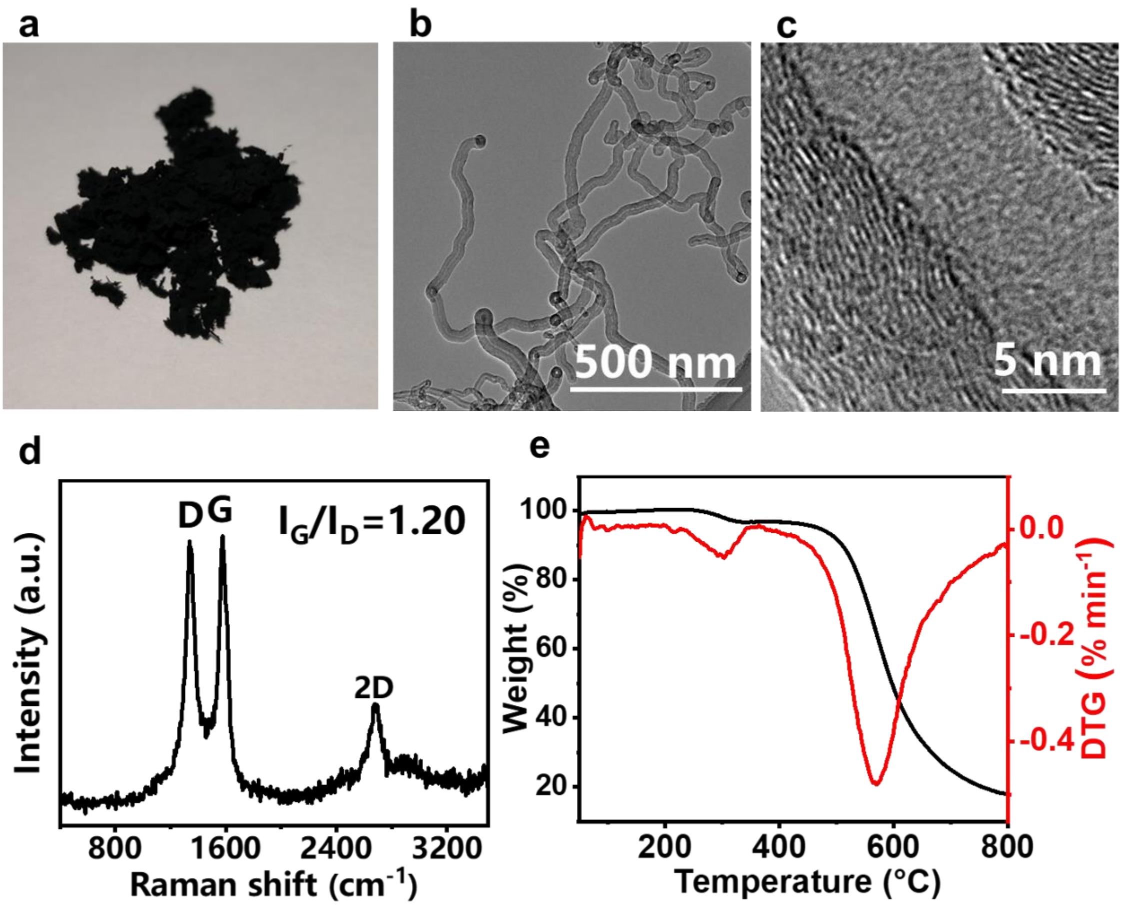

Supplementary Figure 14. CNT synthesis with excessive sulfur. a Digital image and **b, c** TEM images of the curled CNTs. **d** Raman spectrum and **e** TGA of the amorphous CNTs prepared with excessive thiophene addition.

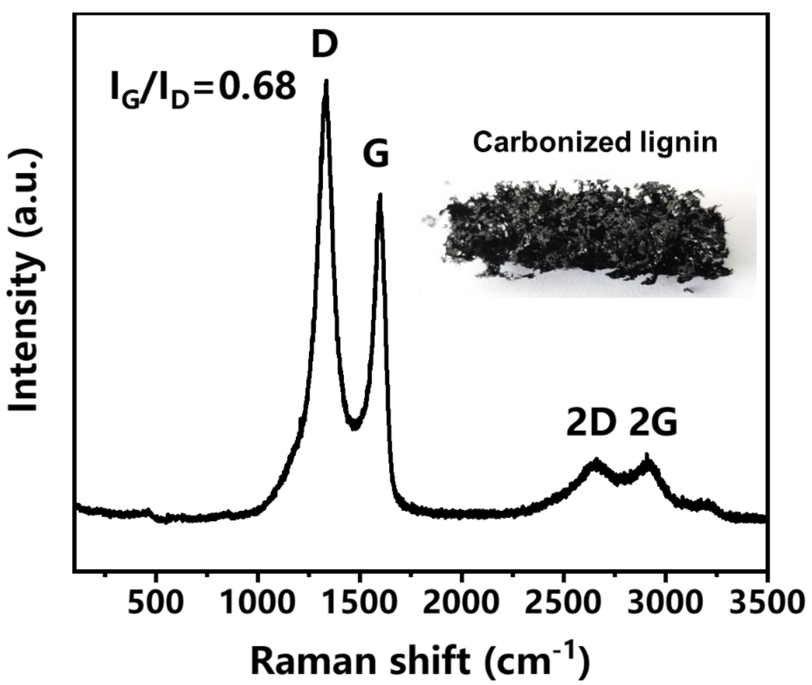

Supplementary Figure 15. Raman spectrum of black charcoal from the pyrolysis of solid lignin at 1400°C for 30 min in N_2 atmosphere.

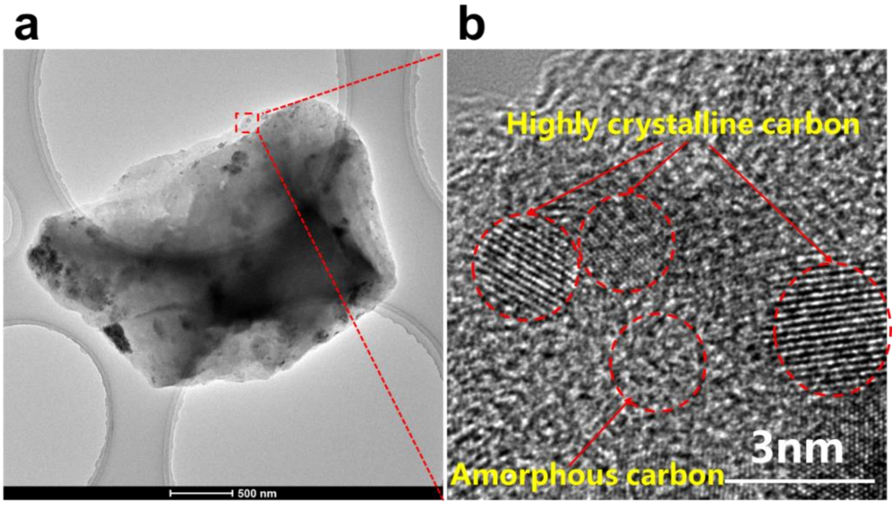

Supplementary Figure 16. Morphologies of the lignin-derived black charcoal. a TEM image and **b** enlarged view of black charcoal from the pyrolysis of solid lignin at 1400°C for 30 min in N_2 atmosphere.

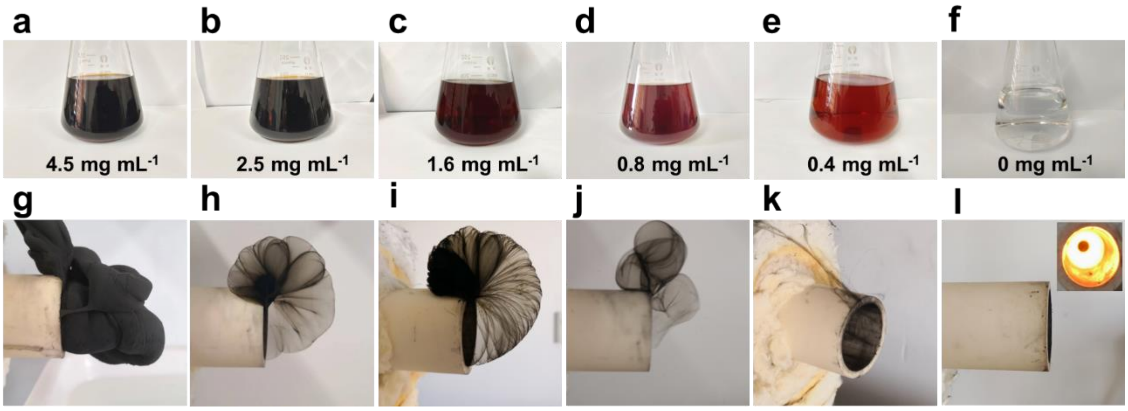

Supplementary Figure 17. Effect of lignin concentration on the synthesis of CNTs. Digital images of lignin solutions with concentrations of **a** 5.5 mg mL^{-1}, **b** 2.5 mg mL^{-1}, **c** 1.6 mg mL^{-1}, **d** 0.8 mg mL^{-1}, **e** 0.4 mg mL^{-1}, **f** 0 mg mL^{-1}. Digital images showing the synthesis of CNTs from lignin solutions with concentrations of **g** 5.5 mg mL^{-1}, **h** 2.5 mg mL^{-1}, **i** 1.6 mg mL^{-1}, **j** 0.8 mg mL^{-1}, **k** 0.4 mg mL^{-1}, **l** 0 mg mL^{-1}.

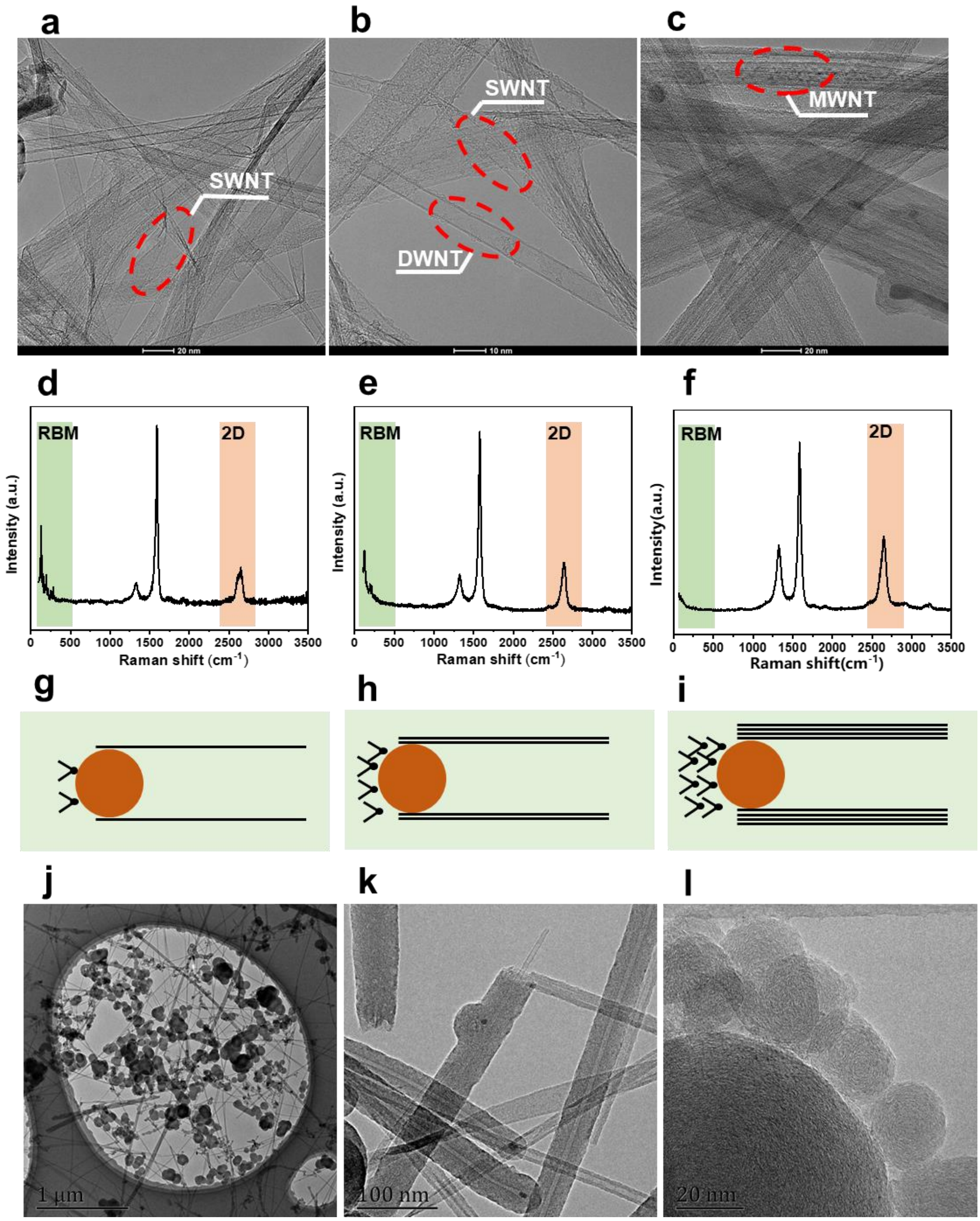

Supplementary Figure 18. Effect of lignin concentration on the morphologies of CNTs. a TEM image, **d** Raman spectrum, **g** growth mechanism of the CNTs prepared with lignin concentration of 0.4 mg mL^{-1}. **b** TEM image, **e** Raman spectrum, **h** growth mechanism of the CNTs prepared with lignin concentration of 0.8 mg mL^{-1}. **c** TEM image, **f** Raman spectrum, **i** growth mechanism of the CNTs prepared with lignin concentration of 2.5 mg mL^{-1}. TEM

images of the **j** CNT aggregates prepared with lignin concentration of 5.5 mg mL^{-1}, and enlarged images of **k** carbon nanorods and **l** amorphous carbon spheres.

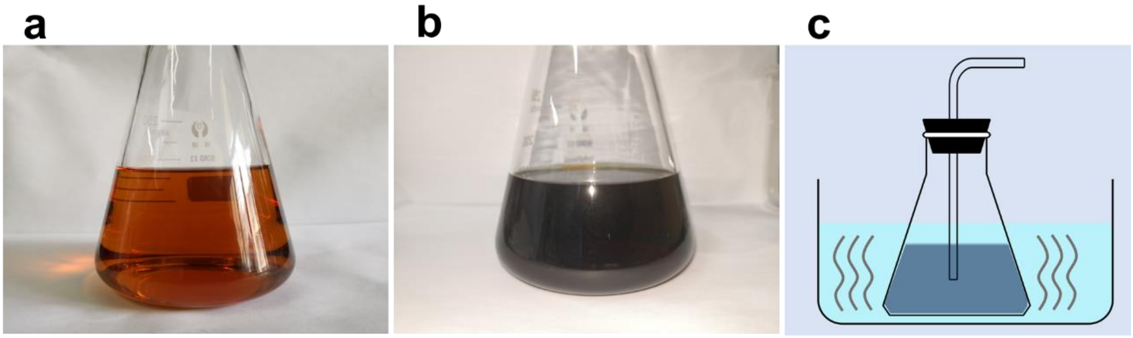

Supplementary Figure 19. Digital images of lignin solutions with a concentration of **a** 0.5 mg mL^{-1}, and **b** 6 mg mL^{-1}. **c** Schematic showing the oscillation of high-concentration lignin solutions during CNT synthesis.

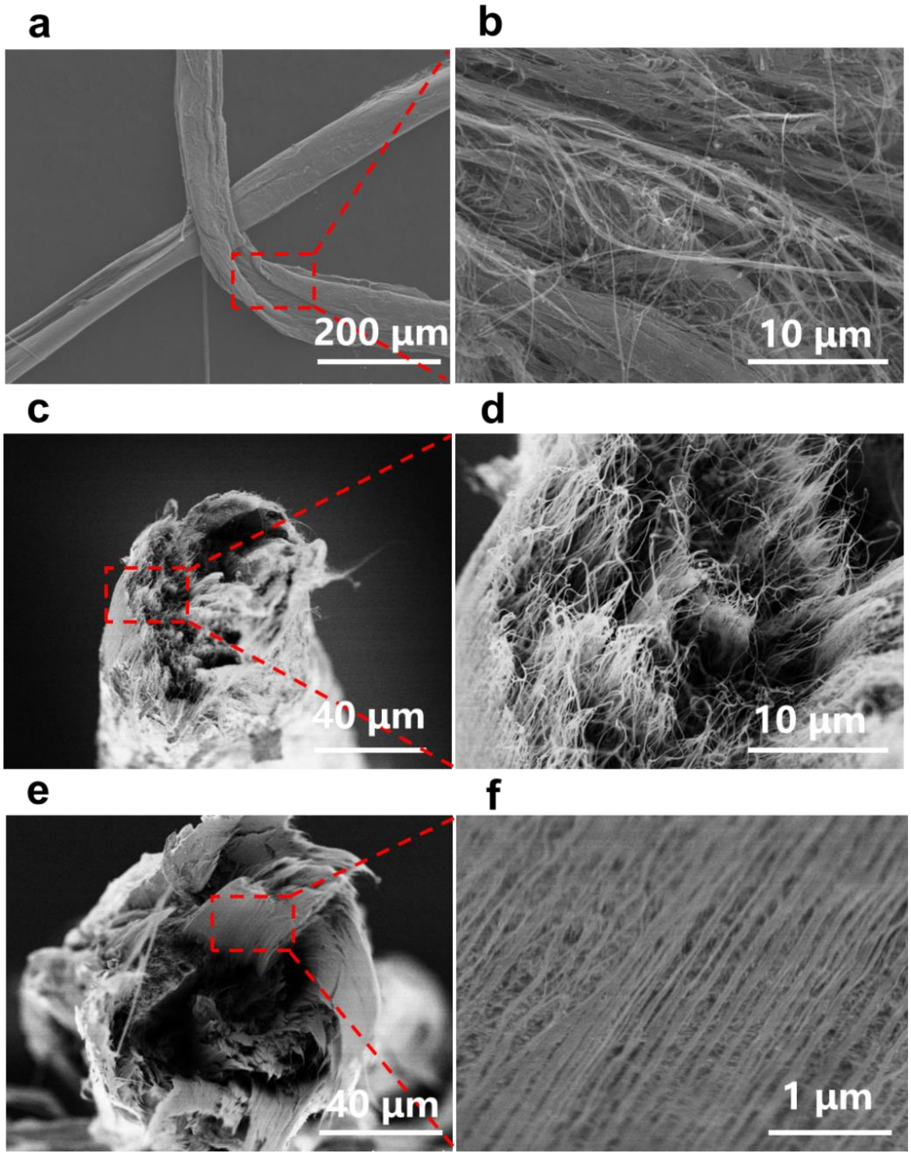

Supplementary Figure 20. Morphologies of the nascent CNT fibers, twisted CNT fibers and rolled CNT fibers. **a** SEM image of the CNT aggregate and **b** magnified SEM image. **c** Cross-sectional SEM image of the twisted CNT fibers and **d** magnified SEM image. **e** Cross-sectional SEM image of the rolled CNT fibers and **f** magnified SEM image.

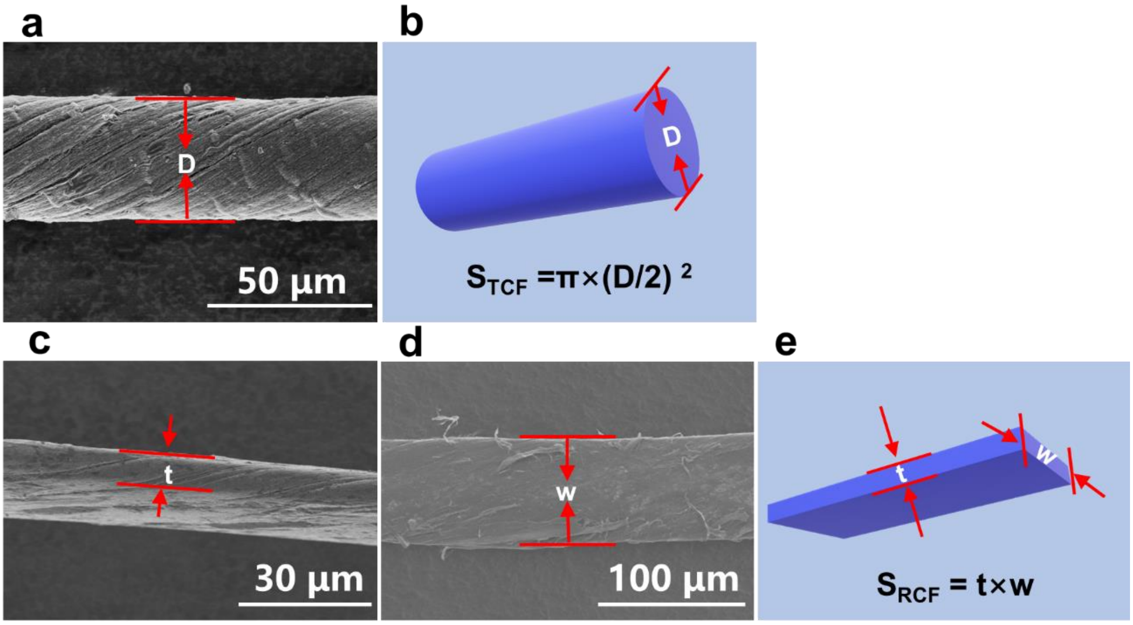

Supplementary Figure 21. a SEM image and **b** calculation model of the TCFs. **c, d** SEM images and **e** calculation model of the RCFs.

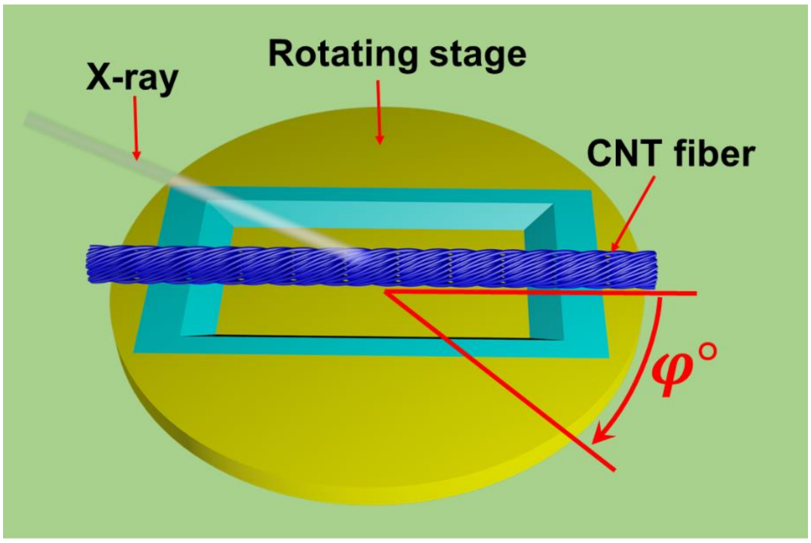

Supplementary Figure 22. Schematic diagram for the determination of the fiber orientation by WAXD azimuthal scanning.

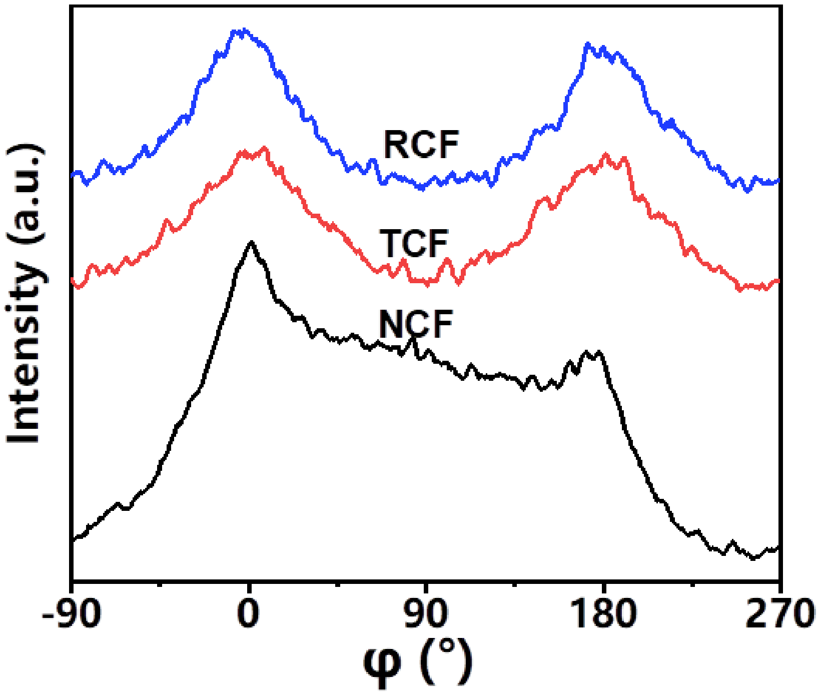

Supplementary Figure 23. WAXD azimuthal scanning of the NCFs, TCFs and RCFs.

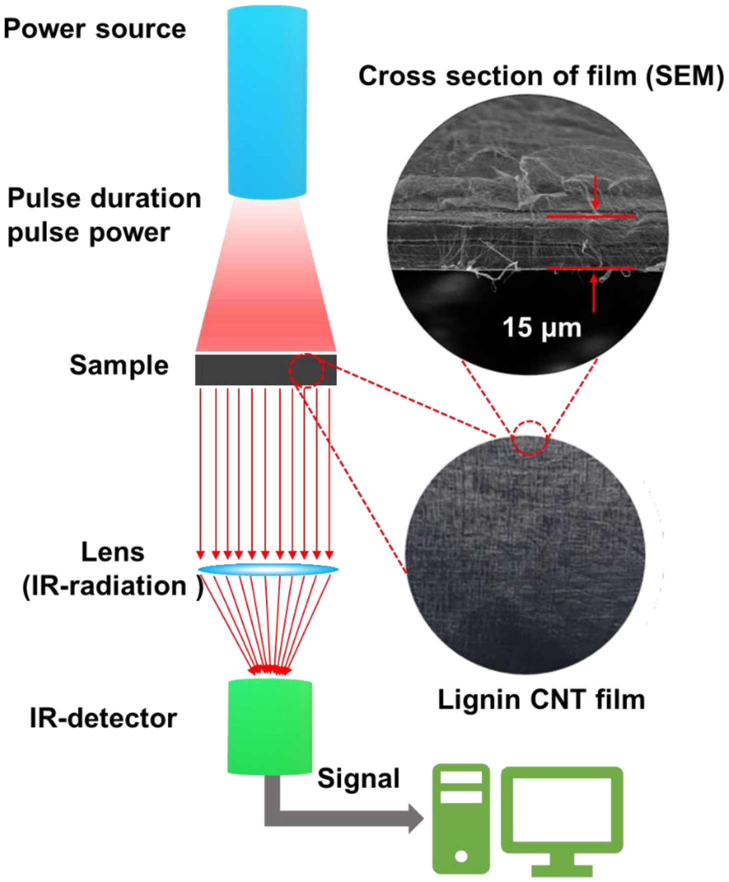

Supplementary Figure 24. Schematic showing the thermal conductivity measurement of the CNT films.

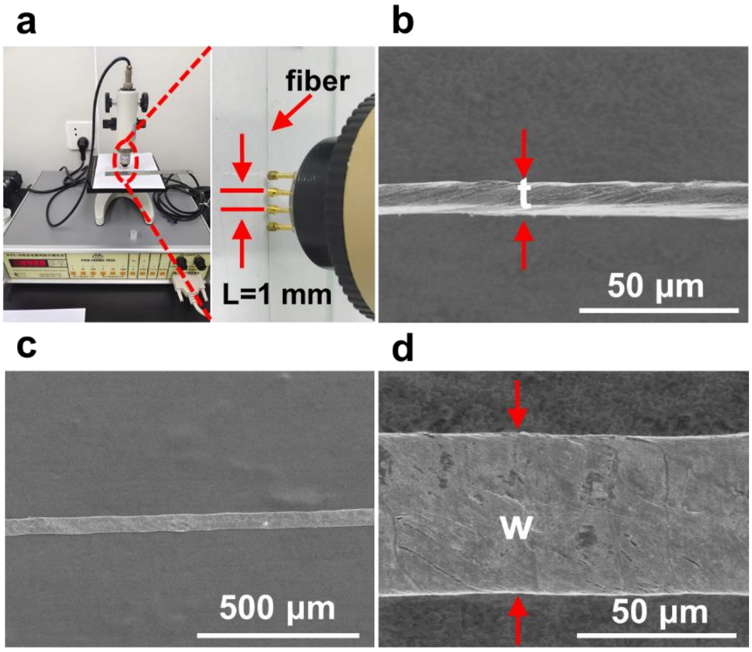

Supplementary Figure 25. Electrical conductivity test of the CNT fibers using four-probe method. **a** Digital image showing the electrical conductivity measurement by four-probe method. **b** SEM image of the CNT fibers to show the thickness (t). **c, d** SEM images of the CNT fibers to show the width (w).

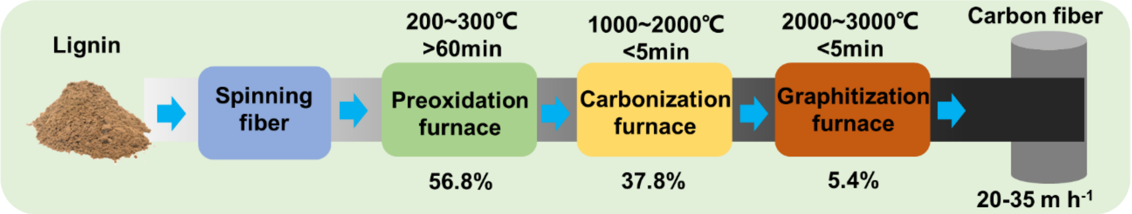

Production rate, m h^{-1}	Energy consumption, MJ	Energy consumption, MJ h^{-1}	Energy consumption, MJ h^{-1}	Energy consumption, MJ h^{-1}	Efficiency, MJ m^{-1}
20	-	7.64	5.08	0.72	0.67
25	-	6.11	4.07	0.58	0.43
30	-	5.09	3.39	0.48	0.30
35	-	4.36	2.90	0.41	0.22

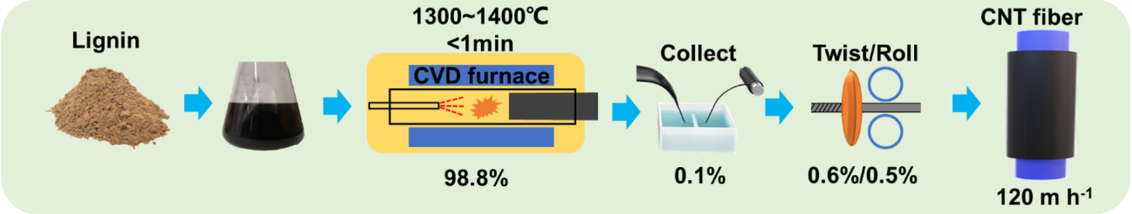

Production rate, m h^{-1}	Energy consumption, MJ	Energy consumption, MJ h^{-1}	Energy consumption, MJ h^{-1}	Energy consumption, MJ h^{-1}	Efficiency, MJ m^{-1}
120	-	14.40	0.02	0.09/0.07	0.12

Supplementary Figure 26. Comparison of energy consumption between lignin-based CNT fibers prepared by FCCVD method in our work and lignin-based carbon fibers prepared by conventional spinning method [63, 64].

Note: For the preparation of carbon fibers, the high-temperature processing part includes pretreatment, carbonization and graphitization, and the data of its energy consumption referred to the research report of Deakin University [63, 64]. For the preparation of our CNT fibers, the energy consumption is mainly in the pyrolysis part of lignin. The pyrolysis of lignin is carried out in a tubular furnace (GSL-1400X, Hefei kejing Material Technology Co., Ltd., China) with a power of 4 KW, which consumes at most 14.4 MJ of energy per hour. For the post-treatment process of CNT fibers, the energy consumption is mainly concentrated in fiber collection, twisting and rolling. An optical axis motor (2GN-18K-50K, rated power=6 W) from Taizhou Weichuang Electromechanical Equipment Co., Ltd. was used for fiber collection, and the energy consumption was 0.02 MJ h^{-1}. A yarn twist meter (Y331A, rated power≤25 W) from Changzhou Yifangyi Spinning Instrument Co., Ltd. was used for fiber twisting, and the energy

consumption was 0.09 MJ h^{-1}. A small automatic roll-to-roll machine (MSK-HRP-04-RD, maximum power=20 W) from Hefei Kejing Material Technology Co., Ltd. was used for fiber rolling, and the energy consumption was 0.07 MJ h^{-1}.

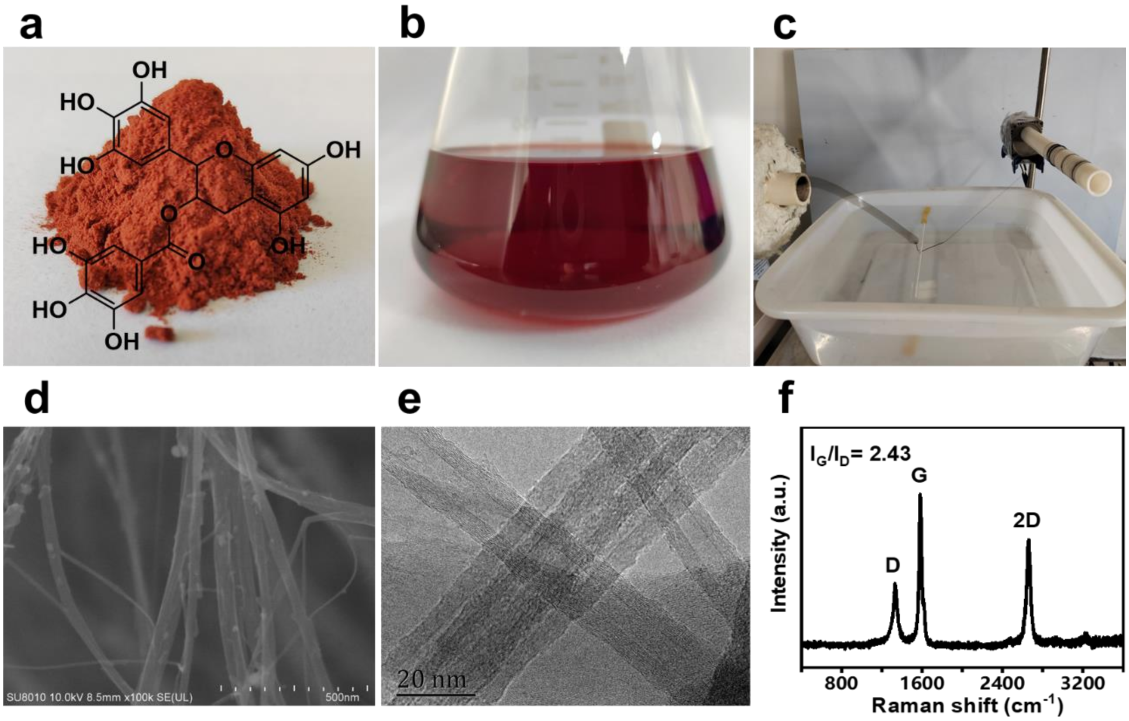

Supplementary Figure 27. Preparation and structures of CNT fibers from tea polyphenols. Digital images of **a** tea polyphenol powder and **b** its methanol solution. **c** Digital image showing the continuous preparation of CNT fibers from tea polyphenols. **d** SEM image, **e** TEM image and **f** Raman spectrum of the CNTs.

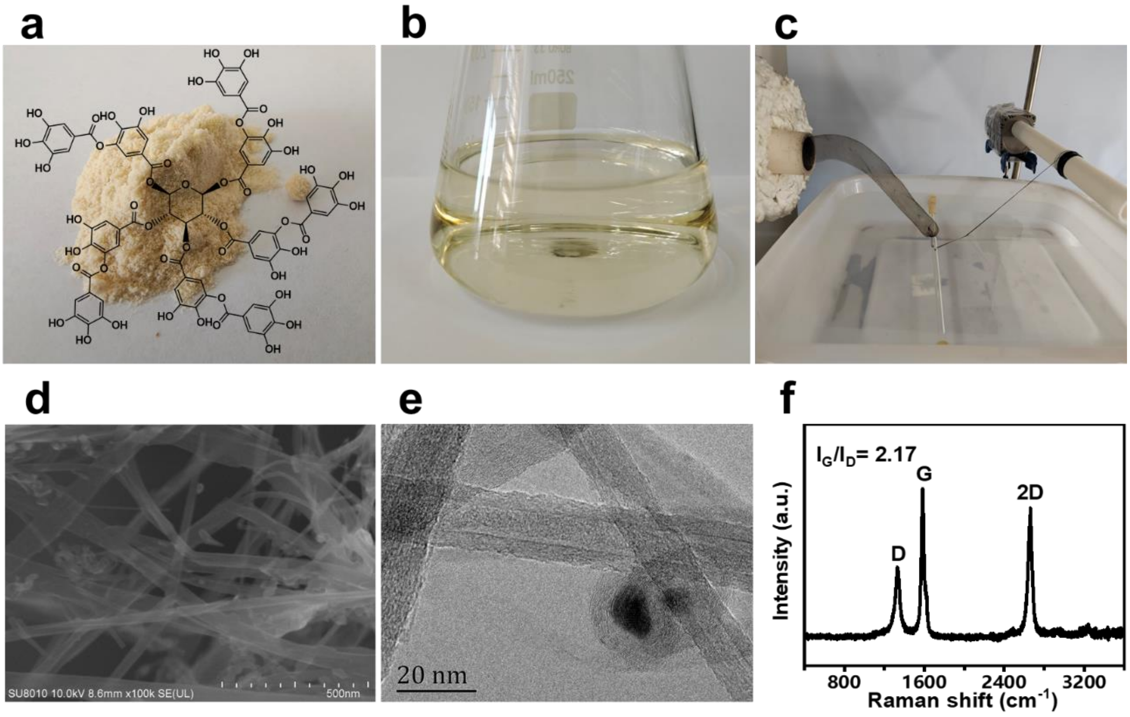

Supplementary Figure 28. Preparation and structures of CNT fibers from tannic acid. Digital images of **a** tannic acid powder and **b** its methanol solution. **c** Digital image showing the continuous preparation of CNT fibers from tannic acid. **d** SEM image, **e** TEM image and **f** Raman spectrum of the CNTs.

Supplementary References

1. Ghosh, P., Afre, R. A., Soga, T. & Jimbo, T. A simple method of producing single-walled carbon nanotubes from a natural precursor: eucalyptus oil. *Mater. Lett.* **61**, 3768-3770 (2007).
2. Ghosh, P. et al. Vertically aligned carbon nanotubes from natural precursors by spray pyrolysis method and their field electron emission properties. *Appl. Phys.* **94**, 51-56 (2009).
3. Kumar, R., Singh, R. K. & Tiwari, R. Growth analysis and high-yield synthesis of aligned-stacked branched nitrogen-doped carbon nanotubes using sesame oil as a natural botanical hydrocarbon precursor. *Mater. Des.* **94**, 166-175 (2016).
4. Kang, Z., Wang, E., Mao, B., Su, Z., Chen, L. & Xu, L. Obtaining carbon nanotubes from grass. *Nanotechnology* **16**, 1192 (2005).
5. Xie, X. et al. A method for producing carbon nanotubes directly from plant materials. *Forest. Prod. J.* **59**, 26–28 (2009).
6. Qu, J., Cong, Q., Luo, C. & Yuan, X. Adsorption and photocatalytic degradation of bisphenol a by low-cost carbon nanotubes synthesized using fallen leaves of poplar. *RSC Adv.* **3**, 961-965 (2013).
7. Lee, J. et al. Direct spinning and densification method for high-performance carbon nanotube fibers. *Nat. Commun.* **10**, 1-10 (2019).
8. Hou, G. F. et al. Gas phase pyrolysis synthesis of carbon nanotubes at high temperature. *Mater. Des.* **132**, 112-118 (2017).
9. Wang, J. N., Luo, X. G., Wu, T. & Chen, Y. High-strength carbon nanotube fibre-like ribbon with high ductility and high electrical conductivity. *Nat. Commun.* **5**, 3848 (2014).
10. Paukner, C. & Koziol, K. K. Ultra-pure single wall carbon nanotube fibres continuously spun without promoter. *Sci. Rep.* **4**, 1-7 (2014).

11. Watanabe, T. et al. Post-synthesis treatment improves the electrical properties of dry-spun carbon nanotube yarns. *Carbon* **185**, 314-323 (2021).

12. Abdullah, H. B., Irmawati, R., Ismail, I. & Yusof, N. A. Direct synthesis of carbon nanotube aerogel using floating catalyst chemical vapor deposition: effect of gas flow rate. *Chem. Pap.* **74**, 3359-3365 (2020).

13. Yang, X. et al. Enhanced catalytic activity of carbon nanotubes for the oxidation of cyclohexane by filling with Fe, Ni, and FeNi alloy nanowires. *Aust. J. Chem.* **69**, 689-695 (2015).

14. Lee, S. H. et al. Synthesis of carbon nanotube fibers from carbon precursors with low decomposition temperatures using a direct spinning process. *Carbon* **124**, 219-227 (2017).

15. Ding, E. X. et al. Highly conductive and transparent single-walled carbon nanotube thin films from ethanol by floating catalyst chemical vapor deposition. *Nanoscale* **9**, 17601-17609 (2017).

16. Khabushev, E. M., Krasnikov, D. V., Kolodiazhnaia, J. V., Bubis, A. V. & Nasibulin, A. G. Structure-dependent performance of single-walled carbon nanotube films in transparent and conductive applications. *Carbon* **161**, 712-717 (2020).

17. Moon, S. Y., Kim, B. R., Park, C. W., Lee, S. H. & Kim, S. M. High-crystallinity single-walled carbon nanotube aerogel growth: Understanding the real-time catalytic decomposition reaction through floating catalyst chemical vapor deposition. *Chem. Eng. J. Adv.* **10**, 100261 (2022).

18. Lee, S. H. et al. Deep-injection floating-catalyst chemical vapor deposition to continuously synthesize carbon nanotubes with high aspect ratio and high crystallinity. *Carbon* **173**, 901-909 (2021).

19. Romanov, S. A., Alekseeva, A. A., Khabushev, E. M., Krasnikov, D. V. & Nasibulin, A. G. Rapid, efficient, and non-destructive purification of single-walled carbon nanotube

films from metallic impurities by Joule heating. *Carbon* **168**, 193-200 (2020).

20. Hong, W.-T. & Tai, N.-H. Investigations on the thermal conductivity of composites reinforced with carbon nanotubes. *Diamond Relat. Mater.* **17**, 1577-1581 (2008).
21. Kim, H. S., Jang, J.-u., Yu, J. & Kim, S. Y. Thermal conductivity of polymer composites based on the length of multi-walled carbon nanotubes. *Composites, Part B* **79**, 505-512 (2015).
22. Yan, Q., Arango, R., Li, J. & Cai, Z. Fabrication and characterization of carbon foams using 100% Kraft lignin. *Mater. Des.* **201**, 109460 (2021).
23. Liu, J. et al. Thermal conductivity and annealing effect on structure of lignin-based microscale carbon fibers. *Carbon* **121**, 35-47 (2017).
24. Wang, R. et al. Anisotropic thermal conductivities and structure in lignin-based microscale carbon fibers. *Carbon* **147**, 58-69 (2019).
25. Li, Y., Fu, Q., Rojas, R., Yan, M., Lawoko, M. & Berglund, L. Lignin-retaining transparent wood. *ChemSusChem* **10**, 3445-3451 (2017).
26. Grishechko, L. I., Amaral-Labat, G. i., Szczurek, A., Fierro, V., Kuznetsov, B. N. & Celzard, A. Lignin-phenol-formaldehyde aerogels and cryogels. *Microporous Mesoporous Mater.* **168**, 19-29 (2013).
27. Zhan, H., Chen, Y. W., Shi, Q. Q., Zhang, Y., Mo, R. W. & Wang, J. N. Highly aligned and densified carbon nanotube films with superior thermal conductivity and mechanical strength. *Carbon* **186**, 205-214 (2022).
28. The Online Materials Information Resource, http://www.matweb.com/.
29. Wang, L. et al. Conductive carbon microfibers derived from wet-spun lignin/nanocellulose hydrogels. *ACS Sustainable Chem. Eng.* **7**, 6013-6022 (2019).
30. Nowak, A. P. et al. Lignin-based carbon fibers for renewable and multifunctional lithium-ion battery electrodes. *Holzforschung* **72**, 81-90 (2018).

31. Li, Q. et al. Microstructure defines the electroconductive and mechanical performance of plant-derived renewable carbon fiber. *Chem. Commun.* **55**, 12655-12658 (2019).

32. Prauchner, M. J., Pasa, V. M. D., Otani, S. & Otani, C. Biopitch-based general purpose carbon fibers: processing and properties. *Carbon* **43**, 591-597 (2005).

33. Kinumoto, T., Matsumura, T., Yamaguchi, K., Matsuoka, M., Tsumura, T. & Toyoda, M. Material processing of bamboo for use as a gas diffusion layer in proton exchange membrane fuel cells. *ACS Sustainable Chem. Eng.* **3**, 1374-1380 (2015).

34. Torres-Canas, F. et al. Improved structure and highly conductive lignin-carbon fibers through graphene oxide liquid crystal. *Carbon* **163**, 120-127 (2020).

35. Wang, Q., Wang, C., Zhang, M., Jian, M. & Zhang, Y. Feeding single-walled carbon nanotubes or graphene to silkworms for reinforced silk fibers. *Nano Lett.* **16**, 6695-6700 (2016).

36. Xu, G., Zhao, J., Li, S., Zhang, X., Yong, Z. & Li, Q. Continuous electrodeposition for lightweight, highly conducting and strong carbon nanotube-copper composite fibers. *Nanoscale* **3**, 4215-4219 (2011).

37. Zhao, J. et al. Vibration-assisted infiltration of nano-compounds to strengthen and functionalize carbon nanotube fibers. *Carbon* **101**, 114-119 (2016).

38. Zhang, X. et al. Ultrastrong, stiff, and lightweight carbon-nanotube fibers. *Adv. Mater.* **19**, 4198-4201 (2007).

39. Zhang, X. et al. Strong carbon-nanotube fibers spun from long carbon-nanotube arrays. *small* **3**, 244-248 (2007).

40. Zhu, H., Xu, C., Wu, D., Wei, B., Vajtai, R. & Ajayan, P. Direct synthesis of long single-walled carbon nanotube strands. *Science* **296**, 884-886 (2002).

41. Tran, T. Q. et al. Purification and dissolution of carbon nanotube fibers spun from the floating catalyst method. *ACS Appl. Mater. Interfaces* **9**, 37112-37119 (2017).

42. Zhao, Y., Wei, J., Vajtai, R., Ajayan, P. M. & Barrera, E. V. Iodine doped carbon nanotube cables exceeding specific electrical conductivity of metals. *Sci. Rep.* **1**, 1-5 (2011).
43. Tran, T. Q., Fan, Z., Liu, P., Myint, S. M. & Duong, H. M. Super-strong and highly conductive carbon nanotube ribbons from post-treatment methods. *Carbon* **99**, 407-415 (2016).
44. Ericson, L. M. et al. Macroscopic, neat, single-walled carbon nanotube fibers. *Science* **305**, 1447-1450 (2004).
45. Behabtu, N. et al. Strong, light, multifunctional fibers of carbon nanotubes with ultrahigh conductivity. *Science* **339**, 182-186 (2013).
46. Tsentalovich, D. E. et al. Influence of carbon nanotube characteristics on macroscopic fiber properties. *ACS Appl. Mater. Interfaces* **9**, 36189-36198 (2017).
47. Taylor, L. W. et al. Improved properties, increased production, and the path to broad adoption of carbon nanotube fibers. *Carbon* **171**, 689-694 (2021).
48. Liu, Y. & Kumar, S. Recent progress in fabrication, structure, and properties of carbon fibers. *Polym. Rev.* **52**, 234-258 (2012).
49. Miao, M. Production, structure and properties of twistless carbon nanotube yarns with a high density sheath. *Carbon* **50**, 4973-4983 (2012).
50. Lee, S.-H., Park, J., Moon, S. Y., Lee, S. Y. & Kim, S. M. Strong and highly conductive carbon nanotube fibers as conducting wires for wearable electronics. *ACS Appl. Nano Mater.* **4**, 3833-3842 (2021).
51. Alemán, B., Reguero, V., Mas, B. & Vilatela, J. J. Strong carbon nanotube fibers by drawing inspiration from polymer fiber spinning. *ACS nano* **9**, 7392-7398 (2015).
52. Zhou, T. et al. The synergetic relationship between the length and orientation of carbon nanotubes in direct spinning of high-strength carbon nanotube fibers. *Mater. Des.* **203**, 109557 (2021).

53. Lee, S.-H. et al. Deep-injection floating-catalyst chemical vapor deposition to continuously synthesize carbon nanotubes with high aspect ratio and high crystallinity. *Carbon* **173**, 901-909 (2021).

54. Reguero, V., Alemán, B., Mas, B. & Vilatela, J. J. Controlling carbon nanotube type in macroscopic fibers synthesized by the direct spinning process. *Chem. Mater.* **26**, 3550-3557 (2014).

55. Song, J., Kim, S., Yoon, S., Cho, D. & Jeong, Y. Enhanced spinnability of carbon nanotube fibers by surfactant addition. *Fibers Polym.* **15**, 762-766 (2014).

56. Karaeva, A. R., Kazennov, N. V., Zhukova, E. A. & Mordkovich, V. Z. Carbon nanotubes by continuous growth, pulling and harvesting into big spools. *Mater. Today: Proc.* **5**, 25951-25955 (2018).

57. Mas, B. et al. Group 16 elements control the synthesis of continuous fibers of carbon nanotubes. *Carbon* **101**, 458-464 (2016).

58. Chauhan, D., Pujari, A., Zhang, G., Dasgupta, K., Shanov, V. N. & Schulz, M. J. Effect of a metallocene catalyst mixture on CNT yield using the FC-CVD process. *Catalysts* **12**, 287 (2022).

59. Shang, Y. Y., Wang, Y., Li, S. H., Hua, C. F., Zou, M. C. & Cao, A. Y. High-strength carbon nanotube fibers by twist-induced self-strengthening. *Carbon* **119**, 47-55 (2017).

60. Xiang, R. et al. Encapsulation, compensation, and substitution of catalyst particles during continuous growth of carbon nanotubes. *Adv. Mater.* **19**, 2360-2363 (2007).

61. Yadav, M. D. & Dasgupta, K. Kinetics of carbon nanotube aerogel synthesis using floating catalyst chemical vapor deposition. *Ind. Eng. Chem. Res.* **60**, 2187-2196 (2021).

62. Othman, R. N. & Wilkinson, A. N. The effects of ferrocene concentration on CNT growth on micron silica gel. *AIP Publishing LLC* **1901**, 020003 (2017).

63. Golkarnarenji, G., Naebe, M., Badii, K., Milani, A. S., Jazar, R. N. & Khayyam, H.

Support vector regression modelling and optimization of energy consumption in carbon fiber production line. *Comput. Chem. Eng.* **109**, 276-288 (2018).

64. Khayyam, H. et al. Improving energy efficiency of carbon fiber manufacturing through waste heat recovery: a circular economy approach with machine learning. *Energy* **225**, 120113 (2021).
