# Supplementary material for: Continuously processing waste lignin into high-value carbon nanotube fibers
Source: Nat Commun. 2022 Sep 30;13:5755. doi: 10.1038/s41467-022-33496-2 (PMC9525656; doi:10.1038/s41467-022-33496-2)
Supplement: Supplementary file 3 — Description of Additional Supplementary Files [file 41467_2022_33496_MOESM3_ESM.pdf]

Description of Additional Supplementary Files

File Name: Supplementary Movie 1
Description: Continuously processing waste lignin into high-value carbon nanotube fibers
